# External validation of clinical decision rules for children with wrist trauma

**DOI:** 10.1007/s00247-017-3787-z

**Published:** 2017-02-28

**Authors:** Marjolein A. M. Mulders, Monique M. J. Walenkamp, Bente F. H. Dubois, Annelie Slaar, J. Carel Goslings, Niels W. L. Schep

**Affiliations:** 1 Trauma Unit, Department of Surgery, Academic Medical Center, Meibergdreef 9, 1105 AZ Amsterdam, The Netherlands; 2 Department of Radiology, Academic Medical Center, University of Amsterdam, Amsterdam, The Netherlands; 3 Department of Surgery, Maasstad Hospital, Rotterdam, The Netherlands

**Keywords:** Adolescent, Child, Decision rule, Distal radius, Fracture, Trauma, Wrist

## Abstract

**Background:**

Clinical decision rules help to avoid potentially unnecessary radiographs of the wrist, reduce waiting times and save costs.

**Objective:**

The primary aim of this study was to provide an overview of all existing non-validated clinical decision rules for wrist trauma in children and to externally validate these rules in a different cohort of patients. Secondarily, we aimed to compare the performance of these rules with the validated Amsterdam Pediatric Wrist Rules.

**Materials and methods:**

We included all studies that proposed a clinical prediction or decision rule in children presenting at the emergency department with acute wrist trauma. We performed external validation within a cohort of 379 children. We also calculated the sensitivity, specificity, negative predictive value and positive predictive value of each decision rule.

**Results:**

We included three clinical decision rules. The sensitivity and specificity of all clinical decision rules after external validation were between 94% and 99%, and 11% and 26%, respectively. After external validation 7% to 17% less radiographs would be ordered and 1.4% to 5.7% of all fractures would be missed. Compared to the Amsterdam Pediatric Wrist Rules only one of the three other rules had a higher sensitivity; however both the specificity and the reduction in requested radiographs were lower in the other three rules.

**Conclusion:**

The sensitivity of the three non-validated clinical decision rules is high. However the specificity and the reduction in number of requested radiographs are low. In contrast, the validated Amsterdam Pediatric Wrist Rules has an acceptable sensitivity and the greatest reduction in radiographs, at 22%, without missing any clinically relevant fractures.

## Introduction

In children, distal radius fractures comprise 25–36% of all fractures [1, 2] and are therefore the most common fractures in children [3]. This high prevalence is most likely a result of the relative weakness of the metaphyseal bone, which has not yet modelled in children [4]. For reasons not clarified, the incidence of distal forearm fractures has shown a significant increase over the last few decades, from 151 in Sweden and 309 in the USA per 100,000 person-years to respectively 240 and 409 per 100,000 person-years [1, 5]. This is accompanied by an increasing number of emergency department visits and requested radiographs, and consequently rising health care costs [1, 6, 7].

The decision whether to request a radiograph of the wrist can be difficult for physicians [8]. Slaar et al. [9] showed that 51% of 1,233 children with a trauma of the wrist who presented at the emergency department of three Dutch hospitals had sustained a wrist fracture. The remaining 49% of the radiographs did not reveal a fracture of the wrist and were potentially unnecessary [9]. The introduction of a clinical decision rule could help to avoid unnecessary radiographs of the wrist and therefore decrease costs and waiting times [10]. For ankle injuries, the Ottawa Ankle Rules was successfully introduced in 1992, showing a 7.2% to 16% reduction in radiographs of the ankle in children since validation [11, 12]. Moreover, this rule has shown a 36-min decrease in length of the emergency department visit in adults [13], along with cost savings of $614,226 to $3,145,910 USD per 100,000 patients [14].

The development of a clinical decision rule consists of three steps: (1) derivation of the rule; (2) (external) validation and (3) implementation of the rule in clinical practice to test its impact on the decision-making of physicians [15]. The second step, validation, is most reliable when it is performed external, in a different population and performed by a different research group than the group who developed the rule [16–18].

Several attempts have been made to generate a clinical decision rule for children with acute wrist trauma in an effort to support physicians in making a more validated decision on whether a wrist radiograph should be acquired. One of these decision rules is the Amsterdam Pediatric Wrist Rules [19]. The Amsterdam Pediatric Wrist Rules is a clinical decision rule that aids in determining the need for a radiograph of the wrist in children, based on age and variables visible deformation, swelling of the distal radius, bone tenderness of the distal radius and the anatomical snuff box, and painful supination. Up till now this is the only rule that has been externally validated, with a sensitivity of 95.9% (95% confidence interval [CI]: 91.7–98.0%) and a specificity of 37.3% (95% CI: 31.0–44.1%).

The primary aim of this study was to provide an overview of all existing non-validated decision rules for wrist trauma in children and to externally validate these rules in a different cohort of patients. Secondarily, we aimed to compare the performance of these rules with the validated Amsterdam Pediatric Wrist Rules.

## Materials and methods

### Selection of existing clinical decision rules for children with wrist trauma

We performed a systematic literature search in Medline (Pubmed) on Dec. 21, 2015, using the search strategy depicted in Table 1. This systematic search was conducted according to the MOOSE (Meta-analysis of observational studies in epidemiology) guidelines [20]. We restricted the languages to English and Dutch. We included all types of studies that proposed a clinical prediction rule or decision rule in children presenting at the emergency department with acute wrist trauma. After screening the title and abstract, we studied full-text articles as to whether the eligible criteria were met. Finally, we performed a cross-reference check. We used the CHARMS checklist for critical appraisal and data extraction [21]. The CHARMS (checklist for critical appraisal and data extraction for systemic reviews of predication modelling studies) checklist has been designed for data extraction and quality assessment for systematic reviews of decision rules. This checklist contains 11 parameters that could lead to bias or that affect the applicability of the results. Two independent authors conducted the data extraction, addressing disagreement with discussion and consensus.

**Table 1:** Search strategy

Search	Items found
((“Wrist Injuries”[Mesh] OR “Arm Injuries”[Mesh] OR wrist injur*[tiab] OR wrist trauma*[tiab] OR wrist[tiab] OR forearm[tiab]) AND (“Child”[Mesh] OR “Adolescent”[MeSH] OR “Pediatrics”[Mesh] OR pediatr*[tiab] OR paediatr*[tiab] OR child*[tiab] OR minor[tiab] OR minors[tiab] OR adolescen*[tiab] OR teen*[tiab]) AND (“Decision Support Techniques”[Mesh] OR (decision*[tiab] AND (rule*[tiab] OR aid*[tiab] OR support*[tiab])) OR clinical decision*[tiab] OR clinical prediction*[tiab]))Filters: English; Dutch	67

### Validation cohort

For external validation we used a study population previously described in the development and external validation study of the Amsterdam Pediatric Wrist Rules [19]. The study included a total of 379 children between 3 years and 18 years old, presenting with 170 wrist fractures and 209 non-fractures between April 6, 2011, and April 15, 2014, at the emergency department of three non-university teaching hospitals in the Netherlands. All consecutive children between 3 years and 18 years old were included if they had pain or tenderness secondary to acute wrist trauma. Acute trauma was defined as wrist trauma sustained within 72 h before presentation at the emergency department [19].

Using a standardised case record form, the data collected comprised 18 variables including patient characteristics, physical examination, functional testing and grip strength measured with a Baseline Hydraulic Hand Dynamometer (Fabrications Enterprises Incorporated, White Plains, New York, USA) (Table 2). All included patients were physically examined before the radiographs were taken. A fracture of the wrist was defined as fracture or epiphysiolysis of the distal radius or the distal ulna, or both [19]. Because fractures of the carpal bones in children are rare and frequently occult on plain radiographs, these fractures were not taken into account [2, 22, 23]. A fracture was recorded if a disruption of one or more cortices of the bone were present. Buckle fractures of bowing fractures were also defined as a true fractures, as were fissures and avulsions.

**Table 2:** Clinical variables of validation cohort

Clinical variables	Missing variables, number of patients (%)
Sex	-
Age	-
Swelling of distal radius	1 (0.1)
Swelling of distal ulna	32 (4.2)
Swelling of anatomical snuffbox	2 (0.3)
Visible deformation	0
Bone tenderness	-
Distal radius	2 (0.3)
Distal ulna	3 (0.4)
Anatomical snuffbox	3 (0.4)
Active mobility painful	-
Dorsiflexion	3 (0.4)
Palmar flexion	4 (0.5)
Supination	3 (0.4)
Pronation	3 (0.4)
Ulnar deviation	4 (0.5)
Radial deviation	5 (0.6)
Functional tests painful^a^	-
Radio ulnar ballottement test^b^	25 (3.2)
Axial compression of forearm	25 (3.2)
Prehensile grip strength^c^	98 (12.5)

^a^Items were scored positive if the patient experienced pain, if they were unable to perform the test or if they refused to perform the test

^b^Test is positive if pain or tenderness occurs when the ulna is translated from volar to dorsal while the radius manually fixated

^c^Both sides assessed three times with a Baseline Hydraulic Hand Dynamometer, expressed in percentage of decrease in grip strength between the healthy and the mean affected side

### Sample size and statistical analysis

The sample for a validation study should include at least 100 events (fractures) and 100 non-events to detect relevant differences [24]. Among such events, a missing value level of less than 5% is considered as an acceptable value to use complete case analysis [25]. Missing completely at random (MCAR) may be used if the missing data are a random sample of the original dataset [26]. During our validation, we used complete case analysis for each decision rule if data were missing completely at random. To determine this, we performed a Little’s MCAR test. If this test was not statistically significant, the data were missing completely at random and complete case analysis could be performed.

We calculated the sensitivity and specificity of each decision rule, as well as the negative and positive predictive values and the 95% confidence intervals. Additionally, we determined the reduction in radiographs requested and the missed fractures rates. We analysed data using SPSS version 22.0 (IBM, Armonk, NY).

## Results

### Study selection

**Fig. 1:**
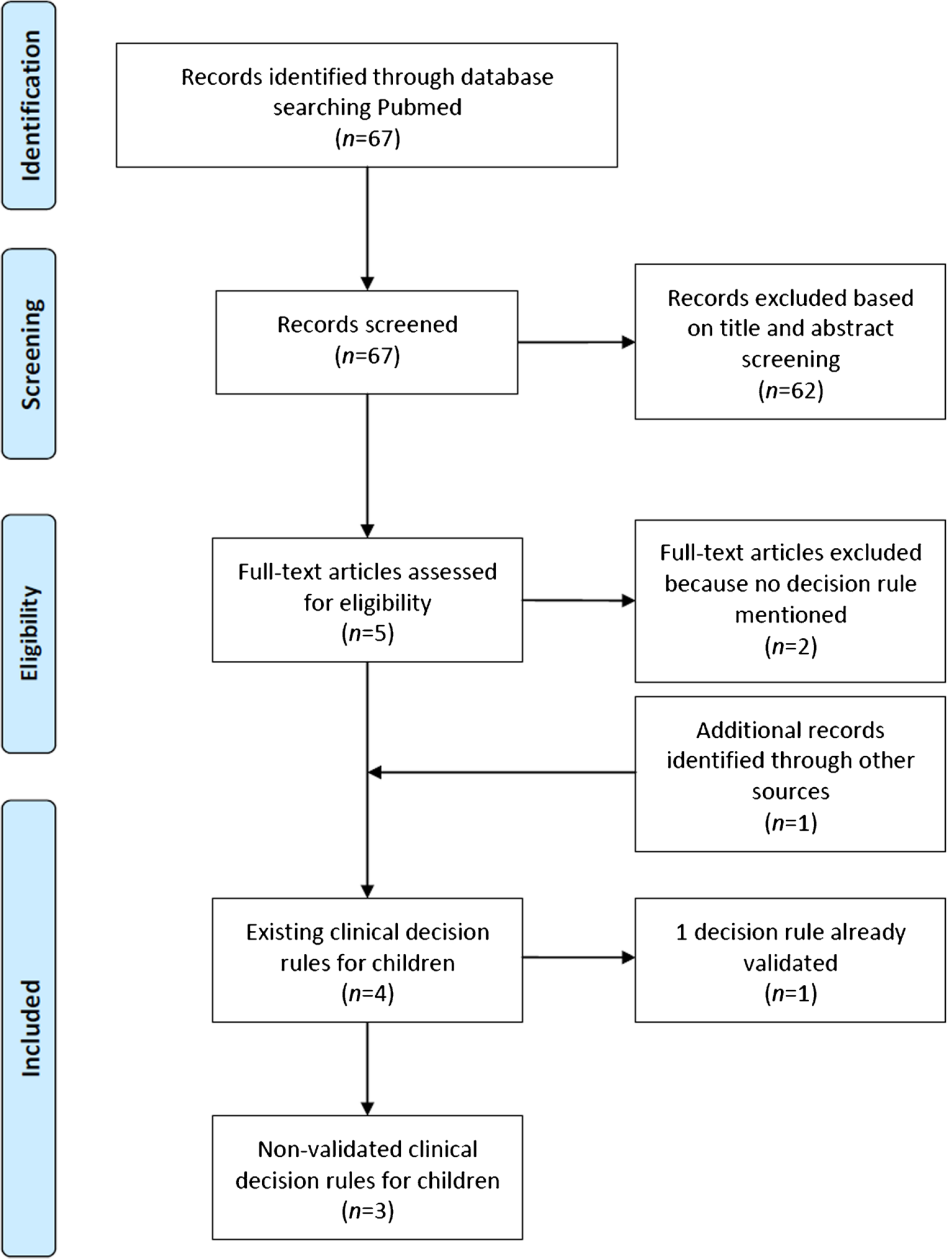
Study selection

The search yielded 67 articles. After title and abstract screening and full-text reading, four articles met all our inclusion criteria (Fig. 1). One of these studies was the previously validated Amsterdam Pediatric Wrist Rules study [19]. This resulted in three other decision rules for children with wrist trauma.

### Study characteristics and results

The first study we included was a prospective blinded case series by Pershad et al. [27]. This study included a total of 48 patients ages 3–18 years who sustained acute wrist injury within the preceding 3 days. This study excluded children with gross deformity at presentation at the emergency department because they were extremely likely to have a fracture. In addition to the standard evaluation after injury, physical examination consisted of measurement of range of motion with a goniometer and measurement of the grip strength with a Martin vigorimeter (Elmed Inc., Addison, IL). Moreover, in each child the zone (distal radius, distal ulna, carpal bones and scaphoid) of maximal tenderness was recorded. A radiograph was obtained in all children after the initial clinical examination. The Wilk log likelihood ratio test was used for the selection of predictors for multivariable modelling [27].

Zone tenderness (*P*=0.005), functional grip strength compared to the uninjured hand (*P*=0.019) and the overall suspicion of a fracture judged by the investigator prior to the radiograph (*P*=0.0083) were all statistically significant predictors. However the authors included only zone tenderness and functional grip strength in their decision rule (Table 3) [27]. The sensitivity of this clinical decision rule in the study cohort was 79% and the specificity was 63% [27]. The negative and positive predictive values were respectively 75% and 68% [27].

The second study was a prospective cohort study of Webster et al. [28]. This study included a total of 227 children ages 3–16 years who presented within 72 h after blunt wrist trauma. Exclusion criteria were patients with gross deformity because of their high likelihood of having sustained a fracture, as well as altered mental status, bone disease and an open fracture. Physical examination included focal swelling; grip strength; zone tenderness; and the range of motion of supination, pronation, dorsiflexion and palmar flexion [28]. To determine zone tenderness, the same zones were used as by Pershad et al. [27]. Additionally, the time between injury and presentation to the emergency department (more or less than 6 h) was taken into account [28]. The decision to request a radiograph of the wrist was at the discretion of the treating physician. All the outcome variables were analysed with the Pearson chi-square test. Subsequently, all variables with *P*<0.2 were included in a multivariate logistic regression model [28].

Univariate analysis showed that 6 of the 10 clinical predictors were significantly associated with a fracture (at a significance level of *P*<0.2). In the multivariate analysis, only radial tenderness (*P*<0.01), reduced supination or pronation (*P*<0.05) and focal swelling (*P*<0.001) were significantly and independently associated with a fracture (Table 3) [28]. This rule had a sensitivity of 99.1% (95% CI: 94.8–100%) and a specificity of 24% (95% CI: 17.2–32.3%) when applied in the study’s own cohort [28].

The third study was a retrospective study by Rivara et al. [29]. This study included a total of 189 children younger than 16 years with an injury that occurred within 7 days prior to the emergency department visit. For each injury, data collected included bone deformity or bone instability, crepitance, pain or limited range of motion, swelling, point tenderness, decreased sensation and ecchymosis. In addition, age, race, sex, time of day and cause and mechanism of injury were recorded. The majority of physicians requested the radiograph before completing the data collection form. However there was no evidence that the timing of completing the data collection form influenced the findings [29].

Before entering variables in a linear discriminant model, odds ratios were calculated for each predictor (entered if p≤0.05). When it was impossible to estimate the relative discriminating power with an odds ratio, because all patients with this symptom had a fracture, a stepwise multivariable discriminant analysis was performed. Gross deformity and point tenderness showed the highest odds ratios, of respectively 16.1 (95% CI: 4.7 to 54.9) and 7.0 (95% CI: 3.2 to 15.6) and were consequently the best discriminators between the fracture and the non-fracture group (Table 3). The presence of ecchymosis was also a significant discriminator. However the presence of ecchymosis was not retained in the decision rule because, in the absence of point tenderness and gross deformity, it did not differ between the fracture and no-fracture groups indicated by the odds ratio [29]. This rule of Rivara et al. [29] showed a sensitivity and specificity of respectively 81% and 82% and negative predictive value of 75% in their own study cohort.

**Table 3:** Decision rules

Pershad et al. [27]	Perform radiograph if both clinical findings are present: 1. Point tenderness over the distal radius 2. Decrease of more than 20% in grip strength compared to the normal hand
Webster et al. [28]	Perform radiograph if at least one of the following clinical findings is present: 1. Radial tenderness 2. Focal swelling 3. Reduction in range of supination and pronation
Rivara et al. [29]	Perform radiograph if at least one of the following clinical findings is present: 1. Gross deformity 2. Point tenderness

### Methodological quality of the studies

All three studies were single-centre studies. None of the studies clarified how missing data were handled. Only Rivara et al. [29] reported the number of missing data for each predictor. Both Pershad et al. [27] and Webster et al. [28] conducted a decision rule for the wrist only, whereas Rivara et al. made one for the whole upper extremity. Additionally, in the study of Rivara et al. not all physicians requested the radiograph after completing the data collection form. Although there was no difference in predictors of positive or negative radiograph findings between data collection forms that were finished after requesting a radiograph and those finished before requesting a radiograph, this could have led to inclusion bias. Last, except for Webster et al. [28], none of the studies mentioned a 95% confidence interval of the sensitivity or specificity, nor the discrimination and calibration curves. For a complete overview of the results of the CHARMS checklist, see Table 4.

**Table 4:** CHARMS checklist for quality assessment

	Pershad et al.	Webster et al.	Rivara et al.
Source of data	Prospective case series	Prospective cohort	Retrospective examination of case records
Participants	Single centre study, Children aged between 3 and 18 years	Single centre study, Children aged between 3 and 16 years	Single centre study, Children less than 16 years
Study dates	Not mentioned	2004, from January 28 to May 14	1984, from Jan 1 to Oct 31
Outcomes and blinding	Fracture of the wrist, physical examination was done before radiographs were taken	Fracture of the wrist, the radiologist was aware of only standard clinical information	Fracture of the upper limb, in some cases the radiograph was taken before physical examination
Follow-up	Phone follow-up was established at day 3–5. If symptoms were persistent or full functional recovery was not obtained, patients were called back to the ED for reevaluation.	Patients who did not have a radiograph were asked to return within 5 days if they still had significant symptoms.	Cases in which the injury was severe were treated conservatively with casting and repeat x-ray films in three to 5 days
Candidate predictors	Measurement of grip strength was done with the Martin vigorimeter. Patients were included <72 h after trauma.	No use of a specific instrument to measure grip strength. Patients were included <72 h after trauma.	The way in which candidate predictors were measured is not mentioned. Patients were included within 7 days after trauma.
Sample size	48 participants, 24 participants with fractures	227 participants, 106 participants with fractures	116 participants, 59 participants with fractures
Missing data	Not mentioned	Not mentioned	Missing values are mentioned, but not the way they were handled.
Model development	Wilks’ log likelihood ratio test was used for detecting associations between the presence of fracture and most predictors. Student’s t-test was used to detect mean value differences in ROM measurements and grip strength.	Univariate variables were analysed with the x2 test. All variables associated with outcome (p,0.2) were entered into a multivariate model (logistic regression) to determine which were independently associated with the outcome.	First odds ratios were determined and after that a linear discriminant model was used for selection of predictors during modelling.
Model performance	Sensitivity 79% Specificity 63% NPV 75% PPV 68%	Sensitivity 99% Specificity 24%	Sensitivity 81% Specificity 82% NPV 75%
Model evaluation	No internal or external validation and no updates	No internal or external validation and no updates	No internal or external validation and no updates
Interpretation and discussion	Prospective validation is needed before we can recommend its adoption.	The low discriminatory value of the rule means that the potential for a clinical decision rule for paediatric wrist trauma appears limited.	The predictive value is low, but could help in the decision making and could lower health care costs.

### External validation

#### Pershad et al. [27]

We evaluated the external performance of the clinical decision rule of Pershad et al. [27] in a cohort of 326 of our 379 subjects. We excluded a total of 53 patients: 28 patients because of the presence of gross deformity and 25 patients after complete case analysis (Little’s MCAR test; *P*=0.337).

The sensitivity and specificity after external validation were respectively 94% (95% CI: 89–97%) and 26% (95% CI: 20–33%). The negative predictive value was 86% (95% CI: 74–93%) and the positive predictive value was 49% (95% CI: 43–55%; Table 5). After applying this clinical decision rule to the validation cohort, 17% less radiographs would have been requested and 5.7% (8) of the fractures would have been missed (Table 6).

**Table 5:** Outcomes after external validation

	External validation in APWR cohort						Original (development) study				
	Sensitivity (95% CI)	Specificity (95% CI)	NPV (95% CI)	PPV (95% CI)	Reduction in requested radiographs	Fractures missed	Sensitivity (95% CI)	Specificity (95% CI)	NPV (95% CI)	PPV (95% CI)	Reduction in requested radiographs
Pershad et al. [27]	94% (89–97%)	26% (20–33%)	86% (74–93%)	49% (43–55%)	17%	5.7%	79%	63%	75%	68%	-
Webster et al. [28]	99% (95–100%)	11% (7–17%)	92% (72–99%)	44% (39–50%)	7%	1.4%	99% (94.8–100%)	24% (17.2–32.3%)	-	-	13%
Rivara et al. [29]	96% (91–98%)	22% (16–28%)	85% (72–93%)	51% (46–57%)	14%	4.3%	81%	82%	75%	-	-
*APWR*	*96*% *(92*–*98*%*)*	*37*% *(31*–*44*%*)*	*92*% *(83*–*96*%*)*	*55*% *(49*–*61*%	*22*%	*4.1*%	*-*	*-*	*-*	*-*	*-*

*APWR* Amsterdam Pediatric Wrist Rules, *CI* confidence interval, *NPV* negative predictive value, *PPV* positive predictive value

**Table 6:** CHARMS (checklist for critical appraisal and data extraction for systemic reviews of predication modelling studies)

	Pershad et al. [27]	Webster et al. [28]	Rivara et al. [29]	*APWR*	Total *(positive by APWR)*
Distal radius	2	0	2	*1*	4 *(5)*
Greenstick	1	1	1	*0*	3 *(3)*
Torus distal radius	3	0	2	*6*	5 *(11)*
Epiphysiolysis distal radius	2	1	2	*0*	5 *(5)*
Radius and ulna	0	0	0	*0*	0 *(0)*
Ulna	0	0	0	*0*	0 *(0)*
Total	8	2	7	*7*	17 *(24)*

*APWR* Amsterdam Pediatric Wrist Rules, *ED* emergency department, *NPV* negative predictive value, *PPV* positive predictive value, *ROM* range of movement

#### Webster et al. [28]

We evaluated the external performance of the clinical decision rule of Webster et al. [28] in a cohort of 351 of our 379 patients. The presence of gross deformity led to the exclusion of 28 subjects. No patients were excluded because of complete case analysis.

The sensitivity in the validation cohort was 99% (95% CI: 95–100%) and the specificity was 11% (95% CI: 7–17%). The negative and positive predictive values were respectively 92% (95% CI: 72–99%) and 44% (95% CI: 39–50%; Table 5). After applying this clinical decision rule to the validation cohort, 7% less radiographs would have been requested and 1.4% (2) of fractures would have been missed (Table 6).

#### Rivara et al. [29]

We evaluated the external performance of the clinical decision rule of Rivara et al. [29] in a cohort of 352 of our 379 patients. We excluded a total of 27 patients because they were 16 years or older. No patients were excluded because of complete case analysis.

The sensitivity and specificity were respectively 96% (95% CI: 91% to 98%) and 22% (95% CI: 16% to 28%). The negative predictive value was 85% (95% CI: 72% to 93%) and the positive predictive value was 51% (95% CI: 46% to 57%; Table 5). After applying this clinical decision rule on the validation cohort, 14% less radiographs would have been requested and 4.3% (7) fractures would have been missed (Table 6).

### Comparison with Amsterdam Pediatric Wrist Rules [19]

The sensitivity of the Amsterdam Pediatric Wrist Rules was 96% (95% CI: 92–98%) and the specificity was 37% (95% CI: 31–44%). This specificity was higher compared to the specificity of the other three rules after external validation. In contrast, the sensitivity was lower compared to the sensitivity of the decision rule of Webster et al. [28] and comparable with the sensitivity of Rivara et al. [29]. However, the Amsterdam Pediatric Wrist Rules showed a 22% reduction of radiographic examinations after external validation, which was higher than the other three decision rules. Although 4.1% of fractures were missed, none of these was clinically relevant.

## Discussion

We included three studies, each describing a non-validated clinical decision rule for children with wrist trauma, and externally validated these rules in the study population in which the Amsterdam Pediatric Wrist Rules was developed and externally validated. The sensitivity of these three clinical decision rules after external validation was high, ranging from 94% to 99%. However, besides a low specificity ranging 11% to 26%, the reduction in radiographs requested without missing any clinically relevant fractures was not of great significance.

In order for physicians to use a clinical decision rule in the emergency department, the sensitivity should be high. Stiell and Wells [30] suggested a sensitivity of at least 96%. This would mean that only the rule of Pershad et al. [27] would not qualify. Conversely, the reduction in radiographs, without missing any clinically relevant fractures, is determinative for the accepted sensitivity. Clinicians might be more willing to use the decision rule if they knew that no fractures would be missed or that any missed fractures would not be clinically relevant. The use of the rule by Webster et al. [28] was accompanied by only 1.4% missed fractures, in contrast with the 5.7% missed fractures in the rule by Pershad et al. [27] and 4.1% in the rule Rivara et al. [29]. However, the rule of Webster et al. [28] only had a reduction in radiograph requests of 7%, which is not of great significance compared to current practice.

Of all 24 missed fractures, 46% were buckle fractures. Buckle fractures are stable fractures and can be treated safely with a soft cast or bandage therapy with good functional outcomes [31–33]. This treatment is equal to the treatment of contusions or sprains of the wrist. Because treatment and prognosis would not have been influenced by a missed or delayed diagnosis [34], these fractures could be considered not clinically relevant. In contrast, after external validation of the other three decision rules, five epiphysiolysis injuries of the distal radius, four extra-articular distal radius fractures and three greenstick fractures were missed. For these missed fractures the clinical impact is considerable and treatment is necessary.

After applying the Ottawa Ankle Rules in children, a pooled reduction of radiographs of approximately 24.8% was observed [35], which is a higher reduction of radiographs than what we found when validating the decision rules for wrist trauma, except for the Amsterdam Pediatric Wrist Rules. This is possibly a result of the very low a priori probability for a fracture in ankle injuries of 14%, compared to 53% for wrist trauma [36, 37]. The higher probability limits the possibilities for improvement. This was confirmed by Van den Brand et al. [36], who investigated the need for a clinical decision rule for patients with blunt wrist trauma. They confirmed the high wrist fracture ratio and recommended radiographs in all patients with wrist trauma presenting at the emergency department. Additionally, they concluded that it is not feasible to develop a decision rule with a high sensitivity and specificity. Despite their conclusion, two fractures in children were missed without even using a clinical decision rule [36]. In contrast, Slaar et al. [9] stated that the development of a clinical decision rule for children with blunt wrist trauma is warranted [9]. Although the costs per radiograph are relatively low, the overall cost of negative radiographs in the three hospitals was approximately €28,608 (U.S. $30,000) per year [9]. A reduction in radiographs could therefore result in cost savings. Furthermore, a reduction in time spent at the emergency department could be realised, like that seen after implementation of the Ottawa Ankle Rules [13].

Compared to the three decision rules that were externally validated in this study, the externally validated Amsterdam Pediatric Wrist Rules showed a 22% reduction of radiographic examinations. At the same time, in external validation this rule missed 4.1% of fractures, although none of these was clinically relevant [19]. The sensitivity of the Amsterdam Pediatric Wrist Rules was 96% (95% CI: 92–98%) and the specificity was 37% (95% CI: 31–44%). This is higher than the specificity of the other three rules after validation. Although the sensitivity of the Amsterdam decision rule is lower than the sensitivity of the Webster et al. [28] decision rule, it is presumably high enough to persuade physicians to use the decision rule in the emergency department.

Looking at the different assessment criteria, except for the 20% decrease in grip strength used in the rule of Pershad et al. [27], the three decision rules used the same variables as in the Amsterdam Pediatric Wrist Rules (i.e. deformity, tenderness of the distal radius, and a reduction in supination). However a possible reason for the better performance of the Amsterdam Pediatric Wrist Rules compared to the other three decision rules is the difference in the derivation of the rule. The Amsterdam Pediatric Wrist Rules uses a linear predictor to calculate the probability of a fracture, whereas the other three rules use the presence or absence of clinical variables. Moreover, age is not taken into account in the three other decision rules.

This study has several limitations. First, we used complete case analysis because only prehensile grip strength had a missing value percentage of more than 5%, namely 12.5%. Because of this complete case analysis, the clinical decision rule of Pershad et al. [27], which contains the variable grip strength, was validated in a smaller cohort than the other two rules. However, the remaining sample was sufficiently large for validation, especially when compared to the size of the derivation cohorts (326 compared to 48 children), and therefore we believe that this does not limit the validity of our results.

Second, we excluded children with gross deformity during the validation of the clinical decision rules by Pershad et al. [27] and Webster et al. [28] because this was one of their exclusion criteria. They excluded these subjects because of their very high likelihood of having a fracture. In general only 86.4% of the children with gross deformity in our dataset, as noted by the emergency department physicians, had a fracture. Although it seems unlikely not to have a fracture with the presence of gross deformity, this could possibly be caused by the swelling that arises after the trauma and mimics a deformity. However, if you remove the evident cases of fractures, it becomes harder for a decision rule to identify fractures and it lowers the sensitivity.

## Conclusion

The sensitivity of the three included clinical decision rules for wrist trauma ranges from 94% to 99%. However, the specificity and the reduction in requested radiographs of these three decision rules are low, and therefore it is doubtful whether these decision rules would supplement current practice. In contrast, the externally validated Amsterdam Pediatric Wrist Rules has been shown to have an acceptable sensitivity and a reduction in radiographs of 22% without missing any clinically relevant fractures.
